# 

**Published:** 1897-07

**Authors:** 


### CONTENTS.

COMMUNICATIONS.

Hygiene of the Teeth ... 313

PROCEEDINGS.

Alabama Dental Association ... 319
Mississippi Dental Association ... 325
South-western Michigan Dental Association ... 334

SELECTIONS.

City Noises Versus Health and Longevity ... 336
Carious Teeth and Tuberculosis ... 340
Extraction of Teeth and Facial Paralysis ... 340
When Pay-Day Comes ... 341

COMMENCEMENTS.

University of Buffalo—Dental Department ... 342
Missouri Dental College ... 342
Birmingham Dental College ... 343
Department of Dental Surgery of the Detroit College of Medicine ... 343
Western Reserve University—Dental College ... 344
College of Dentistry—University of Minnesota ... 344
University College of Medicine—Dental Department, Richmond, Va 345
Dental Department, Southern Medical College, Atlanta, Ga ... 346
Northwesfern College of Dental Surgery ... 346
University of Maryland—Dental Department ... 346
University of California College of Dentistry ... 347
Ohio College of Dental Surgery ... 348
Kansas City Dental College ... 349
Marion Simms College of Medicine—Dental Department ... 449
Baltimore College of Dental Surgery ... 350
University of Pennsylvania ... 350
Howard University—Dental Department ... 351
New York Dental School ... 352
National University—Dental Department ... 352
Westesn University of Pennsylvania—Dental Department ... 353
Boston Dental College ... 353
Columbian University—Dental Department ... 354
Western Dental College ... 354
Atlanta Dental College ... 354
Vanderbilt University ... 355
University of Michigan ... 356
Cincinnati College of Dental Surgery ... 356
Northwestern University Dental School ... 357

NOTICES.

The Southern Dental Association ... 358
The National Association of Dental Examiners ... 359
American Dental Association ... 359
Resolutions of American Academy of Dental Science ... 360

EDITORIAL.

Twelfth International Medical Congress ... 361
Dental Law in Missouri ... 362
Resolutions ... 362
Obituary ... 362
Formaldehyd ... 364

